# HIP/PAP protects against bleomycin‐induced lung injury and inflammation and subsequent fibrosis in mice

**DOI:** 10.1111/jcmm.15334

**Published:** 2020-04-30

**Authors:** Xiaoyan Zheng, Qian Li, Hong Tian, Hanchao Li, Yifei Lv, Yanhua Wang, Lan He, Yongwei Huo, Zhiming Hao

**Affiliations:** ^1^ Department of Rheumatology The First Affiliated Hospital of Xi'an Jiaotong University Xi'an China; ^2^ Research Center of Reproductive Medicine Medical School of Xi'an Jiaotong University Xi'an China; ^3^ Department of Gastroenterology The Third Affiliated Hospital of Xi'an Jiaotong University Xi'an China

**Keywords:** fibrosis, HIP/PAP, inflammation, lung, mouse

## Abstract

Hepatocarcinoma‐intestine‐pancreas/pancreatitis‐associated protein (HIP/PAP), a C‐type lectin, exerts anti‐oxidative, anti‐inflammatory, bactericidal, anti‐apoptotic, and mitogenic functions in several cell types and tissues. In this study, we explored the role of HIP/PAP in pulmonary fibrosis (PF). Expression of HIP/PAP and its murine counterpart, Reg3B, was markedly increased in fibrotic human and mouse lung tissues. Adenovirus‐mediated HIP/PAP expression markedly alleviated bleomycin (BLM)‐induced lung injury, inflammation, and fibrosis in mice. Adenovirus‐mediated HIP/PAP expression alleviated oxidative injury and lessened the decrease in pulmonary superoxide dismutase (SOD) activity in BLM‐treated mice, increased pulmonary SOD expression in normal mice, and HIP/PAP upregulated SOD expression in cultured human alveolar epithelial cells (A549) and human lung fibroblasts (HLF‐1). Moreover, in vitro experiments showed that HIP/PAP suppressed the growth of HLF‐1 and ameliorated the H_2_O_2_‐induced apoptosis of human alveolar epithelial cells (A549 and HPAEpiC) and human pulmonary microvascular endothelial cells (HPMVEC). In HLF‐1, A549, HPAEpiC, and HPMVEC cells, HIP/PAP did not affect the basal levels, but alleviated the TGF‐β1‐induced down‐regulation of the epithelial/endothelial markers E‐cadherin and vE‐cadherin and the over‐expression of mesenchymal markers, such as α‐SMA and vimentin. In conclusion, HIP/PAP was found to serve as a potent protective factor in lung injury, inflammation, and fibrosis by attenuating oxidative injury, promoting the regeneration of alveolar epithelial cells, and antagonizing the pro‐fibrotic actions of the TGF‐β1/Smad signaling pathway.

## 1 INTRODUCTION

Pulmonary fibrosis (PF) is a progressive pathological process characterized by epithelial damage, aberrant proliferation of mesenchymal cells, and the formation of fibrotic foci and is a common outcome of various pulmonary diseases. Numerous factors are involved in its development, including dust, smoking, drugs, infection, auto‐immunity, and radiation. A population of patients with connective tissue diseases was characterized by PF.^1^ Oxidative/antioxidative imbalance^2^, ^3^, ^4^, ^5^, ^6^ and the excessive production of pro‐inflammatory and pro‐fibrotic cytokines^2^, ^3^, ^7^, ^8^, ^9^, ^10^, ^11^ are critically involved in the pathogenesis of PF. Accumulation of myofibroblasts (MFBs) is well recognized as the pivotal event in the PF pathogenesis.^1^ To date, few therapeutic measures have been developed to resolve established PF or hinder PF progression to respiratory failure.^12^ Thus, efforts are required to uncover the mechanisms underlying PF and further explore novel therapeutic targets for this disease.

Hepatocarcinoma‐intestine‐pancreas/pancreatitis‐associated protein (HIP/PAP) is a 16‐kD C‐type lectin that is a member of the regenerating (Reg) protein family. Reg family proteins are classified into four subclasses: types I, II, III, and IV based on their primary structures. HIP/PAP, as well as its counterparts, pancreatitis‐associated protein I (PAP I) in rats and Reg3B in mice, are members of the type III class of the Reg protein family.^13^, ^14^ HIP/PAP shares 52.7% homology in nucleic acid sequence and 70% homology in amino acid sequence with Reg3B. Under physiological conditions, HIP/PAP is secreted by some epithelial cells and certain cells in the pancreas, small intestine, liver, kidney, bladder, pituitary, ovary, and uterus.^13^, ^14^, ^15^, ^16^ HIP/PAP expression was found to be upregulated in inflammatory (pancreatitis, colitis, and hepatitis) and tumor (hepatocellular carcinoma, pancreatic adenocarcinoma, bladder and colorectal carcinoma) tissues.^13^, ^14^, ^15^, ^16^ HIP/PAP has potent mitogenic,^17^, ^18^ bactericidal,^19^ anti‐inflammatory,^17^, ^18^, ^20^, ^21^, ^22^ anti‐oxidative,^17^, ^18^, ^20^, ^21^, ^22^ and anti‐apoptosis^21^, ^22^ functions; thus, it plays extensive and important roles in tissue homeostasis.

In this study, we first measured the expression of HIP/PAP and its mouse counterpart, Reg3B, in human and mouse fibrotic lung tissues. Then, we investigated the effects of recombinant adenovirus‐mediated HIP/PAP expression on bleomycin (BLM)‐induced lung injury, inflammation, and fibrosis in mice and explored the possible underlying mechanisms.

## 2 MATERIALS AND METHODS

### 2.1 Ethics

This study was conducted in accordance with the ethical guidelines of the Helsinki Declaration for experiments involving humans and was approved by the Ethics Committee of the First Affiliated Hospital of Xi'an Jiaotong University (Xi'an, China). All animal experiment protocols were approved by the Institutional Animal Ethics Committee of Xi'an Jiaotong University.

### 2.2 Human specimens

Surgically resected, paraffin‐embedded human fibrotic lung tissue specimens (10 cases) and pathologically normal para‐tumor lung tissue specimens (10 cases) were obtained from the Department of Pathology, the First Affiliated Hospital of Xi'an Jiaotong University, with the approval of the Institutional Review Board. The clinical information of the human specimens is listed in Supplementary Table S1.

### 2.3 Animal experiments

#### 2.3.1 Animals

Specific pathogen‐free, 6‐week‐old male ICR mice, weighing 25‐30 g were provided by the Experimental Animal Center, School of Medicine, Xi'an Jiaotong University. The mice were housed under pathogen‐free conditions under a 12 hours light/dark cycle at constant temperature (22 ± 2°C) and humidity, with free access to water and standard laboratory chow. All mice were acclimatized to the abovementioned conditions for one week before initiating experiments. All efforts were undertaken to minimize the suffering of the mice.

#### 2.3.2 Verification of adenovirus transduction

Replication‐incompetent adenoviruses carrying HIP/PAP (AdHIP/PAP) and green fluorescence protein (AdGFP) were prepared as described previously^18^ using the AdEasy adenovirus vector system (Stratagene, La Jolla, CA, USA). Plaque assays using HEK293 cells were performed to determine the titers of transduced adenoviruses, expressed as plaque‐forming units per milliliter (pfu/mL)**.**

To verify the transduction efficiency of repeated intraperitoneal (i.p.) adenovirus injection, twelve mice were equally assigned to the AdGFP and AdHIP/PAP groups. Mice of each group were given an i.p. injection of AdGFP (5 × 10^9^ pfu) or AdHIP/PAP (5 × 10^9^ pfu) on day 0. Two mice from each group were randomly euthanized on day 7 and day 14, respectively. The remaining mice were injected again with AdGFP (5 × 10^9^ pfu) or AdHIP/PAP (5 × 10^9^ pfu) on day 14 and were sacrificed on day 21. Another two mice were set aside as controls administered an i.p. injection of normal saline (NS) and were euthanized on day 0. The lungs of euthanized mice were harvested for further examination.

Genomic DNA (gDNA) was extracted from lung tissues using a DNeasy Kit (Qiagen, Valencia, CA, USA). One microgram (μg) of the extracted gDNA was used to determine the abundance of the viral vector CMV promoter in lung tissues by PCR amplification.

#### 2.3.3 Adenovirus‐mediated HIP/PAP expression upon bleomycin (BLM)‐induced lung injury and fibrosis

To observe the expression of Reg3B in mouse lung after BLM treatment, thirty mice were equally divided into normal saline (NS, n = 5) and bleomycin (BLM, n = 25) groups. At day 0, the mice were intratracheally (i.t.) instilled with BLM (50 μL, 2.4 mg/mL) or an equal volume of NS as described previously.^23^ Five mice from the BLM group were euthanized on days 3, 7, 14, 21, and 28, while all the mice in the NS group were euthanized on day 3. Mouse lungs were collected for haemotoxylin and eosin (H&E) and picrosirius red staining, western blotting, and other experiments.

To investigate the effects of HIP/PAP on acute lung injury and fibrosis, eighty mice were equally assigned into four groups (n = 20): NS, NS + BLM, AdGFP + BLM, and AdHIP/PAP + BLM. Mice in the two Ad groups were i.p. administered the corresponding recombinant adenovirus (AdGFP or AdHIP/PAP, 5 × 10^9^ pfu in 0.25 mL) for the first time, while mice in the other two groups were dosed with an equal volume of NS. Two days later (Day 0), the mice were i.t. instilled with NS or BLM. Ten mice in each group were sacrificed on day 3. The remaining mice received the second i.p. administration of adenoviruses or NS on day 12 (two weeks after the first adenovirus administration) and were sacrificed on day 28, when the lungs and serum were harvested for subsequent experiments. The mice were weighed during BLM modeling, and their lung coefficient was calculated (lung coefficient = lung wet weight/body weight × 100).

#### 2.3.4 HIP/PAP on SOD expression in mouse lungs

To test the effect of adenovirus‐mediated HIP/PAP expression on SOD expression in mouse lungs, fifteen mice were equally allocated into normal saline (NS), AdGFP, and AdHIP/PAP groups and treated as described above. Seven days later, all mice were euthanized and their lungs were collected. See Section 2.5 for a description of the measurement of SOD expression.

### 2.4 Bronchoalveolar lavage (BAL)

BAL was carried out on day 3 following BLM administration. Immediately after the mice were sacrificed, their lungs and trachea were extracted en bloc, and a 20G intravenous catheter was inserted into their trachea. One milliliter of PBS was instilled into the lungs and withdrawn three times via the catheter. More than 85% of the fluid was recovered as bronchoalveolar lavage fluid (BALF), which was then centrifuged at 1000 rpm for 10 minutes at 4°C. The supernatants were collected and stored at −80°C for protein quantification and ELISA. The precipitate was washed with red blood cell lysis buffer (eBioscience, San Diego, CA, USA) and resuspended in 500 µL of PBS for counting total white blood cells.

### 2.5 Measurement of myeloperoxidase (MPO) and superoxide dismutase (SOD) activities and malondialdehyde (MDA) content

MDA content and MPO and SOD activities in mouse lung tissue and/or cell lysates were determined using commercially available kits (Nanjing Jiancheng Bioengineering Institute, Nanjing, China) in accordance with the manufacturer's protocols. The MDA content was expressed as nmol per milligram protein (nmol/mg prot). MPO activity was expressed as units per gram wet tissue (U/g). SOD activity was expressed as units per milligram protein (U/mg prot). In addition, the possible SOD‐like activities of HIP/PAP and Reg3B were tested by replacing cell lysates with various amounts (3, 6, and 12 ng/well) of recombinant HIP/PAP (rHIP/PAP) and recombinant Reg3B (rReg3B) (both from Sino biological Inc, Beijing, China), respectively.

### 2.6 Measurement of hydroxyproline content

Pulmonary hydroxyproline content was determined using a commercially available kit (Nanjing Jiancheng Bioengineering Institute, Nanjing, China) in accordance with the manufacturer's protocols and expressed as milligrams per gram tissue (mg/gram).

### 2.7 Enzyme‐linked immunosorbent assay (ELISA)

BALF HMGB1, TNF‐α, IL‐1β, and IL‐6 levels were determined by ELISA using commercially available kits (HMGB1 ELISA kit, Chondrex, Redmond, WA, USA; TNF‐α ELISA kit, R&D Systems, Minneapolis, MN, USA; IL‐1β and IL‐6 ELISA kits, eBioscience, San Diego, CA, USA) in accordance with the manufacturer's instructions.

### 2.8 Cell culture and in vitro experiments

#### 2.8.1 Cell lines and culture

A549 (human alveolar epithelial carcinoma cells) and HLF‐1 (human lung fibroblast cells) were purchased from the American Type Culture Collection (ATCC, Rockville, MD, USA), HPAEpiC (human pulmonary alveolar epithelial cells) and HPMVEC (human pulmonary microvascular endothelial cells) was purchased from ScienCell Research Laboratories (ScienCell, CA, USA). A549, HPAEpiC, and HPMVEC were cultured in DMEM medium (Gibco, Grand Island, NY, USA), while HLF‐1 was cultured with F‐12K medium (Gibco, Grand Island, NY, USA) supplemented with 10% fetal bovine serum (Gibco) at 37°C and 5% CO_2_. Three biological replicates and three technical replicates were set for each experiment.

#### 2.8.2 Cell proliferation assay

Cells were inoculated into 96‐well plates at 500 cells per well and allowed to adhere for 24 hours. Then, the complete medium was replaced with DMEM or F‐12K containing 2% FBS and various concentrations of rHIP/PAP (final concentrations of 0, 125, and 250 ng/mL), and cells were cultured for 72 hours. Cell viability was assessed using the cell counting kit‐8 (CCK‐8, Dojindo, Kyushu, Japan) assay at 24, 48, and 72 hours, in accordance with the manufacturer's instructions.

#### 2.8.3 H_2_O_2_‐induced cell injury

A newly opened bottle of H_2_O_2_ solution (10 mol/L) was diluted to 0.1 mol/L with distilled water and added to the culture medium (DMEM or F‐12K containing 2% FBS) to achieve the desired working concentration (0, 200, 400, and 800 μmol/L). Cells were treated with different concentrations of H_2_O_2_ for 12 hours and were then subjected to the CCK‐8 assay.

To test the protective effects of HIP/PAP against H_2_O_2_‐induced injury in lung alveolar epithelial cells and pulmonary microvascular endothelial cells, A549, HPAEpiC, and HPMVEC inoculated into 6‐well plates were assigned to blank control, rHIP/PAP, H_2_O_2_ and rHIP/PAP + H_2_O_2_ groups, and treated accordingly. Cells in the rHIP/PAP and HIP/PAP + H_2_O_2_ groups were pretreated with rHIP/PAP (125 ng/mL) for 2 hours. Then, cells in the two H_2_O_2_ groups were challenged with H_2_O_2_ (200 μmol/L) for 12 hours. Finally, the cells were collected, stained with Annexin V‐fluorescein isothiocyanate (FITC) and propidium iodide (PI) (Merck Millipore, Germany), and analyzed by flow cytometry.

#### 2.8.4 SOD expression

A549 and HLF‐1 cells were seeded into 6‐well plates at a density of 5 × 10^4^ cells per well and allowed to adhere for 24 hours; then, the medium was replaced with serum‐free medium supplemented with rHIP/PAP (125 ng/mL). Seventy‐two hours later, the cells were harvested, lysed with normal saline for assessing SOD activity or with TRIzol for assessing RNA extraction for qRT‐PCR. The culture medium was also used for the determination of SOD activity.

#### 2.8.5 Cell transformation

HLF‐1, A549, HPAEpiC, and HPMVEC cells were seeded into 6‐well plates at a density of 5 × 10^4^ cells per well, allowed to adhere for 24 hours, and divided into the following groups: blank control, rHIP/PAP, rhTGF‐β1, and rhTGF‐β1 + rHIP/PAP. Then, the complete media were replaced with DMEM (for A549, HPAEpiC and HPMVEC) and F‐12K (for HLF‐1) supplemented with 2% FBS. Cells were treated with rHIP/PAP (125 ng/mL) and/or rhTGF‐β1 (5 ng/mL) for 48 hours and then harvested for RNA and protein extraction. Cells grown on coverslips were subjected to immunofluorescence staining.

### 2.9 Quantitative real‐time polymerase chain reaction (qRT‐PCR)

Total RNA was extracted from cultured cells and mouse lung tissues using the TRIzol reagent (Thermo, Life Technologies, Carlsbad, CA, USA). Reverse transcription was performed using the RevertAid First Strand cDNA Synthesis Kit (Thermo Scientific, Rockford, AL, USA). The relative abundance of mRNA in each sample was determined by qRT‐PCR using the SYBR Premix Ex Taq™II kit (TaKaRa, Dalian, China) and specific primers (designed and synthesized by TaKaRa, listed in Supplementary Table S2) on an iQ^TM^ Multicolor Real‐time PCR Detection System (Bio‐Rad, Hercules, CA, USA). Data were analyzed using the ΔΔCT method and β‐actin served as the internal control. The results are presented as mean ± SD of triplicate reactions from three separate experiments.

### 2.10 Western blotting

Total protein was extracted from lung tissues or collected cells, fractionated using sodium dodecyl sulfate‐polyacrylamide gel electrophoresis (SDS‐PAGE), and transferred onto polyvinylidene fluoride (PVDF) membranes (Bio‐Rad, Hercules, CA, USA). The primary antibodies used were mouse anti‐β‐actin mAb (Sigma‐Aldrich, St. Louis, MO, USA), mouse anti‐HIP/PAP pAb (a self‐prepared polyclonal antibody reacts with HIP/PAP, Reg3B and PAPI, as described previously^18^), mouse anti‐GFP pAb (self‐prepared), mouse anti‐SMA mAb (Thermo, Lab Vision, Fremont, CA, USA), mouse anti‐TGF‐β1 mAb (Abcam, Cambridge, UK), rabbit anti‐phospho‐Smad2 pAb (Ser465/467)/Smad3 (Ser423/425) (Cell Signaling Technology, Danvers, MA, USA), and rabbit anti‐Smad2/3 (Cell Signaling Technology).

### 2.11 Histological staining, immunohistochemistry, and immunofluorescence

Formalin‐fixed, paraffin‐embedded mouse lung tissues were subjected to H&E and picro‐sirius red staining to assess pulmonary architectural alterations and collagen deposition. Lung fibrosis was graded in a blinded fashion using previously established criteria.^24^

Immunohistochemistry was carried out using the streptavidin/peroxidase reagent kit (Zymed, San Francisco, CA, USA) in accordance with the manufacturer's protocol. Mouse anti‐α‐SMA mAb (Thermo, Lab Vision, Fremont, CA, USA), mouse anti‐HIP/PAP pAb (self‐prepared), and mouse anti‐CD45 mAb (eBioscience, San Diego, CA, USA) were used as the primary antibodies. Secondary antibody alone was used for the control to exclude nonspecific immunostaining. Computer‐assisted semi‐quantitative analysis using Image‐ProPlus version 4.5 (Media Cybernetics, Silver Spring, MD) was carried out to measure the integrated optical density (IOD) of HIP/PAP‐ or Reg3B‐positive staining. HIP/PAP or Reg3B immunostaining was expressed as an average of the IOD/the total area (excluding empty alveolar cavities and other lumens) from 5 × 200 fields in each immunostained section.

For immunofluorescence staining, cells on the coverslips were fixed in 4% paraformaldehyde for 20 minutes, washed with PBS and permeabilized with 0.1% (v/v) Triton X‐100 for 10 minutes at room temperature, followed by blocking with 10% (v/v) normal goat serum in PBS for 1 hour. Then, the cells were incubated with mouse anti‐α‐SMA mAb (Thermo, Lab Vision, Fremont, CA, USA), rabbit anti‐E‐cadherin mAb (Abcam, Cambridge, MA, USA), rabbit anti‐vE‐cadherin mAb (Abcam), and mouse anti‐vimentin mAb (Abcam) at 4°C overnight. After washing, the slides were incubated with FITC‐ or TRITC‐conjugated secondary antibodies (Thermo, Lab Vision, Fremont, CA, USA). The nuclei were stained with PI (Sigma‐Aldrich, St. Louis, MO, USA) or 4’, 6‐diamidino‐2‐phenylindole (DAPI, Roche, Basel, Switzerland).

### 2.12 Statistical analysis

Statistical analyses were performed using GraphPad Prism software version 6 (GraphPad Software Inc, La Jolla, CA, USA). The data are presented as mean ± standard deviation (SD). Differences among groups were analyzed using one‐way analysis of variance (ANOVA), followed by Tukey's post‐hoc test. *P* < 0.05 was considered statistically significant.

## 3 RESULTS

### 3.1 HIP/PAP (Reg3B) expression was elevated in human and mouse fibrotic lung tissues

**FIGURE 1:**
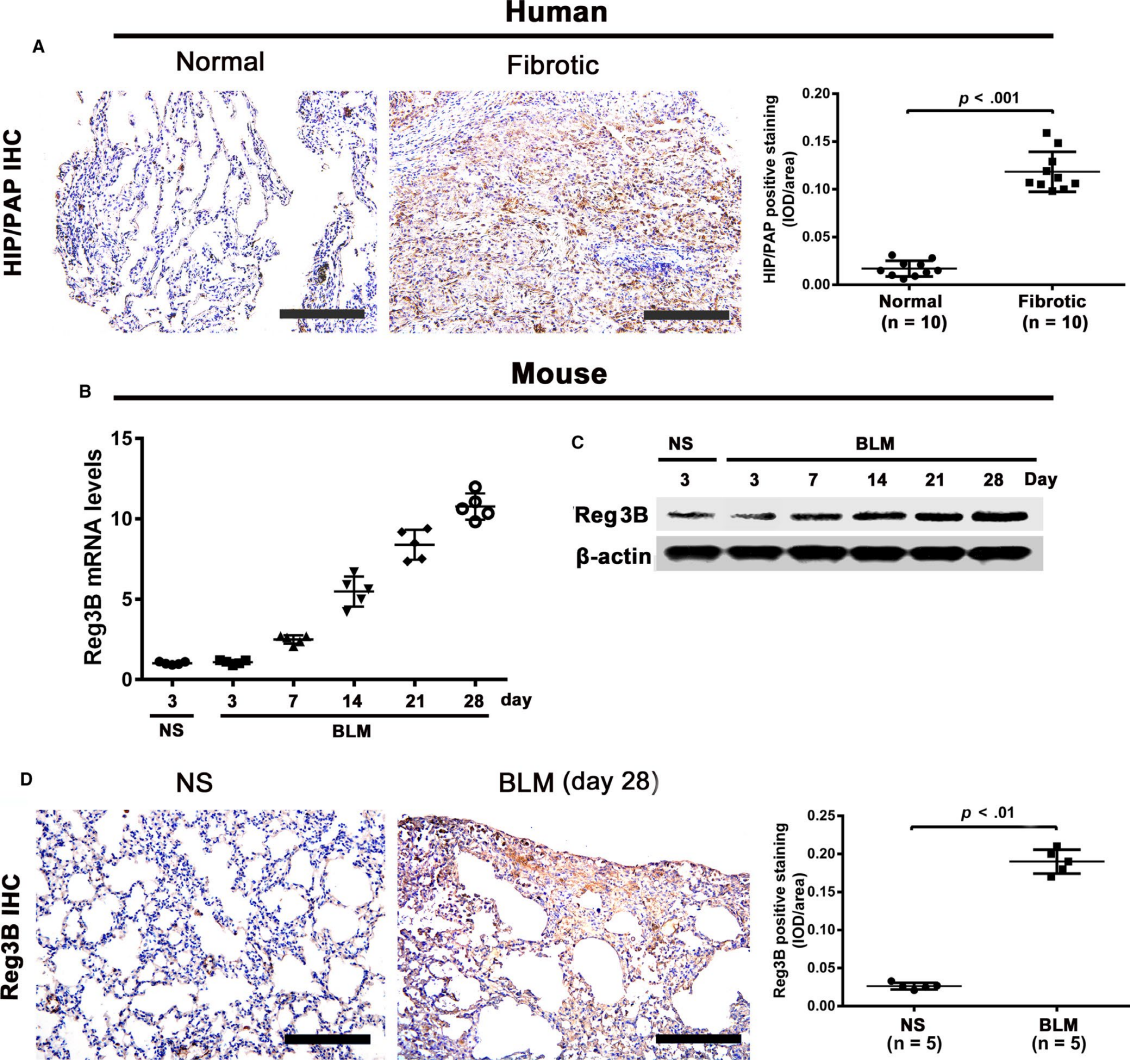
HIP/PAP (Reg3B) expression is elevated in human and mouse fibrotic lung tissues. Immunohistochemistry showed that type II alveolar epithelial cells were positively stained with anti‐HIP/PAP antibody in normal human lung tissues. HIP/PAP expression was drastically elevated in fibrotic human lung tissues, with the majority of the HIP/PAP‐positive signals found in seemingly hyperplastic alveolar epithelial cells (A). Expression of Reg3B in mouse lung tissues was upregulated at both the mRNA (B) and protein (C) levels from day 3 post‐BLM installation and increased steadily until day 28. Immunohistochemical staining showed that the expression patterns of Reg3B in normal and fibrotic mouse lung tissues are similar to those in the normal and fibrotic human lung tissues (D). Error bars indicate SD. Scale bars = 100 μm

Immunohistochemical staining showed that in normal human lung tissues, only a few type II alveolar epithelial cells were positively stained with the anti‐HIP/PAP antibody. Contrarily, in fibrotic human lung tissues, a marked increase in HIP/PAP‐positive cell numbers, the majority of which were seemingly hyperplastic alveolar epithelial cells, led to a significant increase in the average IOD compared with that in normal tissues (Figure 1A). In BLM‐treated mice, qRT‐PCR and western blotting consistently demonstrated that the lung expression of Reg3B was markedly elevated starting from day 7 after BLM treatment and increased thereafter (Figure 1B,C). The increased Reg3B expression in fibrotic mouse lung tissue was also confirmed by immunohistochemistry, which showed that type II alveolar epithelial cells were weakly or moderately immunostained with the anti‐HIP/PAP antibody in normal mouse lung tissues, while numerous hyperplastic epithelial cells were strongly immunostained with the anti‐HIP/PAP antibody in fibrotic mouse lung tissues (Figure 1D). In addition, we tested the effect of TGF‐β1, the pivotal pro‐fibrotic cytokine, on the expression of HIP/PAP in A549 and HLF‐1 cells. TGF‐β1 (5 ng/mL) suppressed HIP/PAP expression at the mRNA level, while HIP/PAP (125 ng/mL) alleviated the TGF‐β1‐induced decrease in HIP/PAP expression in both cell lines (Figure S1).

### 3.2 Intraperitoneal (i.p.) administration of adenovirus efficiently transduced mouse lung tissue

To verify the transduction of recombinant adenoviruses in mouse lungs, the CMV‐IE sequence was detected using PCR. The CMV‐IE sequence was found to be amplified in the lung tissues of AdGFP and AdHIP/PAP mice (Figure S2A). Moreover, western blotting showed that expression levels of HIP/PAP and GFP following the second injection of the recombinant adenoviruses were comparable to those observed following the first injection (Figure S2B). These results clearly indicated that realizing prolonged ectopic expression by repeated i.p. injection of recombinant adenoviruses was feasible. In addition, histological examination, BALF protein concentration determination and cell counting, and lung tissue MPO activity measurement did not reveal any obvious lung injury after either the first or the second administration of the recombinant adenoviruses (data not shown).

### 3.3 HIP/PAP protected mice from BLM‐induced lung injury and inflammation

**FIGURE 2:**
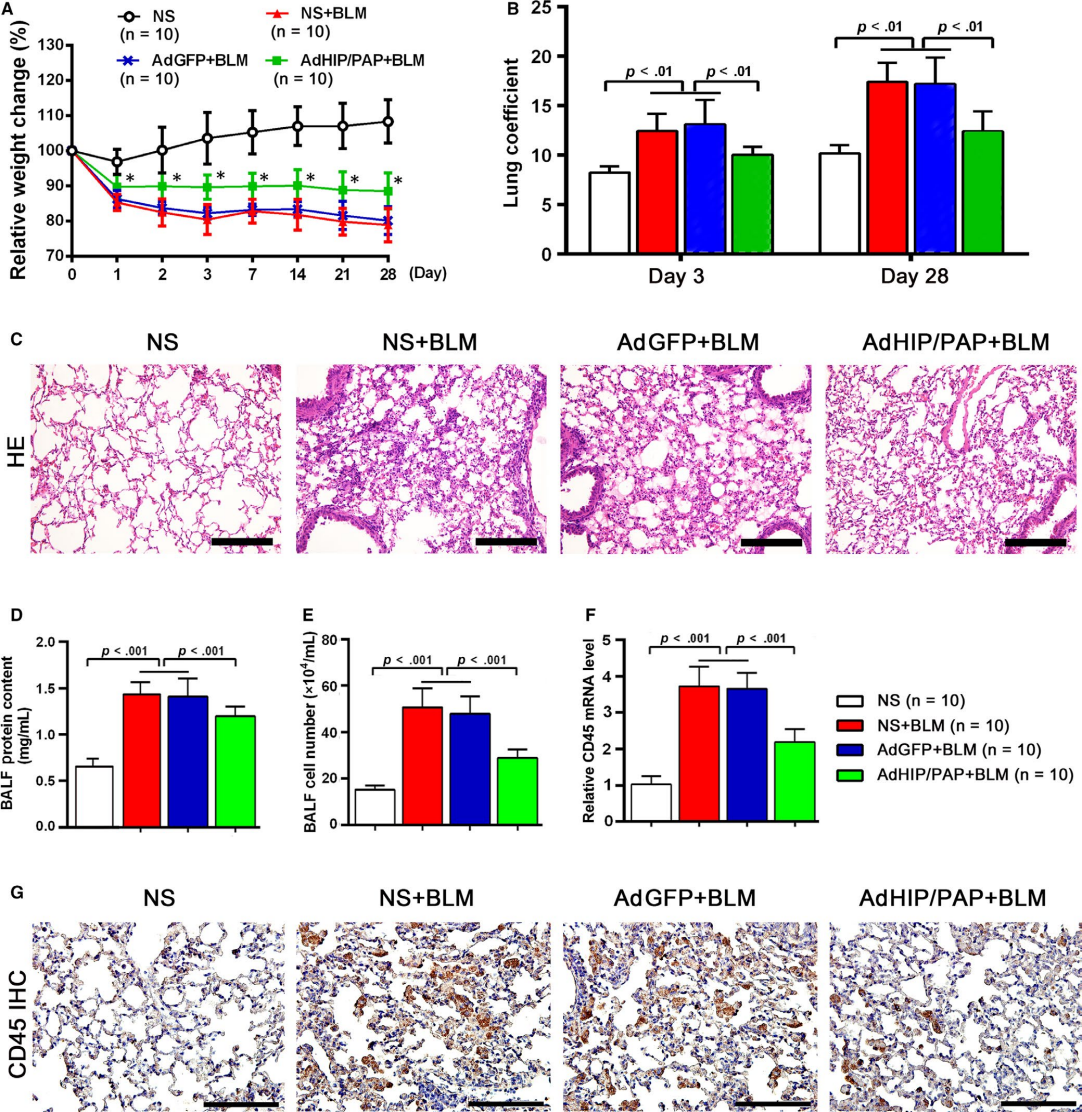
AdHIP/PAP alleviates BLM‐induced lung injury and inflammation in mice. Mice in the AdHIP/PAP + BLM group lost less body weight than those in the other two BLM groups. ^*^ *P* < 0.05 vs the NS + BLM or AdGFP + BLM groups and *P* < 0.01, vs the NS group (A). Similarly, AdHIP/PAP significantly alleviated the increase in the mouse lung coefficient on both day 3 and day 28 after BLM installation (B). Adenovirus‐mediated HIP/PAP expression obviously mitigated lung injury and inflammation, as indicated by H&E staining of mouse lungs (C), BALF protein concentration determination (D), and cell counting (E) on day 3 after BLM instillation consistently showed that the anti‐inflammatory action of HIP/PAP is substantiated by the decreased expression of CD45 in mouse lung, as indicated by qRT‐PCR and immunohistochemistry results (F, G). Error bars indicate SD. Scale bars = 100 μm

The body weights of mice continuously decreased and their lung coefficient was markedly increased after BLM treatment; AdHIP/PAP significantly attenuated these changes (Figure 2A,B). In agreement with previous reports,^17^, ^18^, ^21^, ^22^ HIP/PAP exhibited anti‐oxidative potency, as indicated by lower tissue MDA content in the AdHIP/PAP group than that in the NS + BLM or AdGFP + BLM groups, both on day 3 and day 28 (Table 1). Three days after BLM instillation, histological examination revealed extensive lung injury and inflammation characterized by interstitial edema, leakage of fluid and plasma proteins into the alveolus, infiltration of inflammatory cells, and formation of hyaline membranes, and AdHIP/PAP markedly alleviated these changes in the mouse lung (Figure 2C). Accordingly, BLM‐induced increases in BALF protein content (Figure 2D) and total cell number (Figure 2E) were significantly attenuated by AdHIP/PAP.

**TABLE 1:** AdHIP/PAP alleviates the increases in MDA content and MPO activity in mouse lung tissue after BLM treatment (Mean ± SD)

	Time	NS (n = 10)	NS + BLM (n = 10)	AdGFP + BLM (n = 10)	AdHIP/PAP + BLM (n = 10)
MDA (nmol/mg prot.)	Day 3	0.65 ± 0.67	1.38 ± 0.11	1.44 ± 0.33	0.90 ± 0.19*
	Day 28	0.72 ± 0.33	1.66 ± 0.46	1.58 ± 0.32	1.14 ± 0.28*
MPO (U/g)	Day 3	0.41 ± 0.43	0.87 ± 0.21	0.92 ± 0.34	0.50 ± 0.15*
	Day 28	0.58 ± 0.21	1.03 ± 0.27	0.97 ± 0.13	0.72 ± 0.15*

* *P* < 0.05 vs the NS + BLM or AdGFP + BLM group.

While tissue MPO activity, an indicator of oxidative injury as well as neutrophil infiltration, was elevated by BLM treatment, this increase was also ameliorated by HIP/PAP (Table 1). To further validate the anti‐inflammatory function of HIP/PAP in BLM‐treated mice, amounts of CD45, a leukocyte marker, were measured using qRT‐PCR and immunohistochemistry. The results consistently revealed that ectopic HIP/PAP significantly alleviated the BLM‐induced increase in CD45 expression during the acute phase (Figure 2F,G). Moreover, pre‐treatment with AdHIP/PAP significantly mitigated the increase in the tissue mRNA expression and/or BALF level of inflammatory mediators, such as TNF‐α, IL‐1β, IL‐6, IL‐17, and HMGB1, in fibrotic mouse lungs (Table 2).

**TABLE 2:** AdHIP/PAP abates the BLM‐induced upregulation of pro‐inflammatory factors in mouse lungs

	NS (n = 10)	NS + BLM (n = 10)	AdGFP + BLM (n = 10)	AdHIP/PAP + BLM (n = 10)
mRNA levels (relative fold changes)				
TNF‐α				
Day 3	1.01 ± 0.08	4.82 ± 0.36^#^	4.79 ± 0.38^#^	2.64 ± 0.33*
Day 28	1.00 ± 0.04	2.51 ± 0.31^#^	2.33 ± 0.39^#^	1.46 ± 0.23*
IL‐1β				
Day 3	1.00 ± 0.03	10.40 ± 0.55^#^	10.22 ± 0.70^#^	7.25 ± 0.85*
Day 28	1.00 ± 0.04	3.36 ± 0.14^#^	3.49 ± 0.26^#^	2.51 ± 0.31*
IL‐6				
Day 3	1.00 ± 0.05	10.56 ± 0.60^#^	10.89 ± 0.81^#^	8.07 ± 0.90*
Day 28	1.01 ± 0.04	2.52 ± 0.29^#^	2.45 ± 0.25^#^	1.58 ± 0.27*
IL‐17A				
Day 3	1.01 ± 0.05	5.23 ± 0.25^#^	4.93 ± 0.44^#^	2.86 ± 0.41*
Day 28	1.00 ± 0.06	2.63 ± 0.27^#^	2.63 ± 0.31^#^	2.40 ± 0.26
Concentrations in BALF (pg/mL)				
HMGB1	4.10 ± 0.45	7.46 ± 1.07^#^	7.32 ± 0.86^#^	5.04 ± 0.71*
TNF‐α	6.46 ± 1.87	14.69 ± 1.67^#^	14.77 ± 1.68^#^	9.02 ± 1.97*
IL‐1β	64.19 ± 1.90	82.04 ± 3.33^#^	82.05 ± 4.0^#^	69.68 ± 2.55*
IL‐6	75.93 ± 1.25	89.32 ± 7.19^#^	89.77 ± 6.48^#^	79.87 ± 3.75*

^#^ *P* < 0.01 vs the NS group, and

* *P* < 0.01 vs the NS + BLM or AdGFP + BLM group.

**FIGURE 3:**
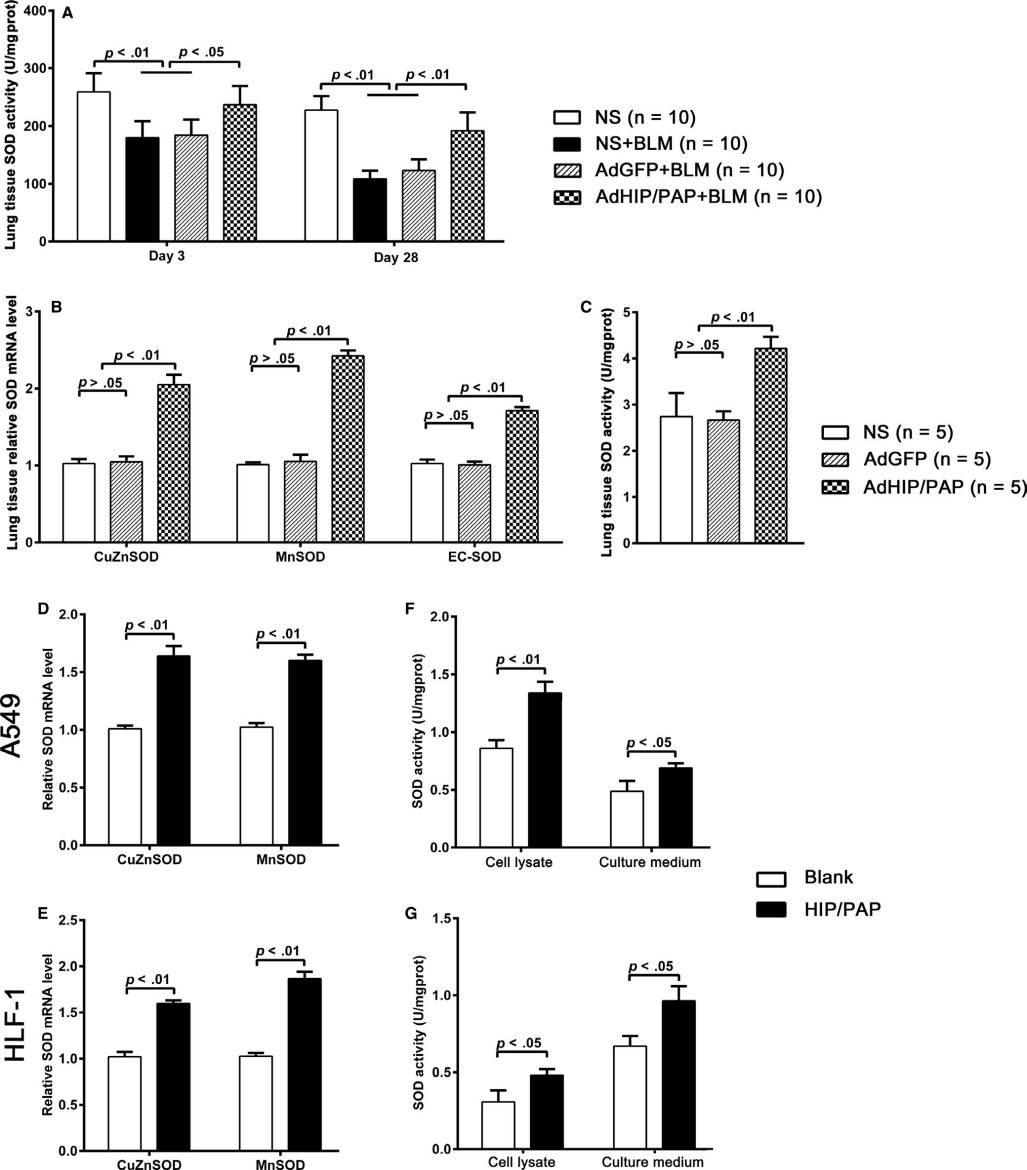
HIP/PAP increases SOD expression and activity in mouse lung tissue as well as cultured A549 and HLF‐1 cells. AdHIP/PAP alleviated the BLM‐induced decrease in SOD activity in mouse lung tissue (A). Moreover, AdHIP/PAP markedly increased SOD (Cu‐Zn SOD, Mn‐SOD and EC‐SOD) mRNA expression (B), resulting in an elevation of total SOD activity in otherwise normal mouse lung tissue (C). Likewise, rHIP/PAP (125 ng/mL) elevated Cu‐Zn SOD and Mn‐SOD mRNA expression in A549 (D) and HLF‐1 (E) cells, as well as total SOD activity in the cell lysates and culture medium (F, G). Error bars indicate SD

Lieu et al proposed that HIP/PAP might exhibit superoxide dismutase‐like and glutathione reductase‐like activities.^17^ In our study, however, HIP/PAP did not exhibit any in vitro SOD‐like activity in a nitro‐blue tetrazolium (NBT) assay (data not shown). We found that the total SOD activity in mouse lung tissue was markedly decreased by BLM treatment and that this decrease was significantly alleviated by AdHIP/PAP administration (Figure 3A). In addition, both SOD (Cu‐Zn SOD, Mn‐SOD and EC‐SOD) mRNA expression and SOD activity in lung tissue were significantly elevated by AdHIP/PAP in otherwise normal mice (Figure 3B,C). Moreover, rHIP/PAP upregulated Cu‐Zn SOD and Mn‐SOD mRNA expression in both A549 and HLF‐1 cells (Figure 3D,E) and increased total SOD activities in their cell lysates and culture medium (Figure 3F,G). These results clearly indicated that HIP/PAP exerts its anti‐oxidative action, at least partially, through the promotion of SOD expression.

### 3.4 HIP/PAP attenuated BLM‐induced lung fibrosis in mice

**FIGURE 4:**
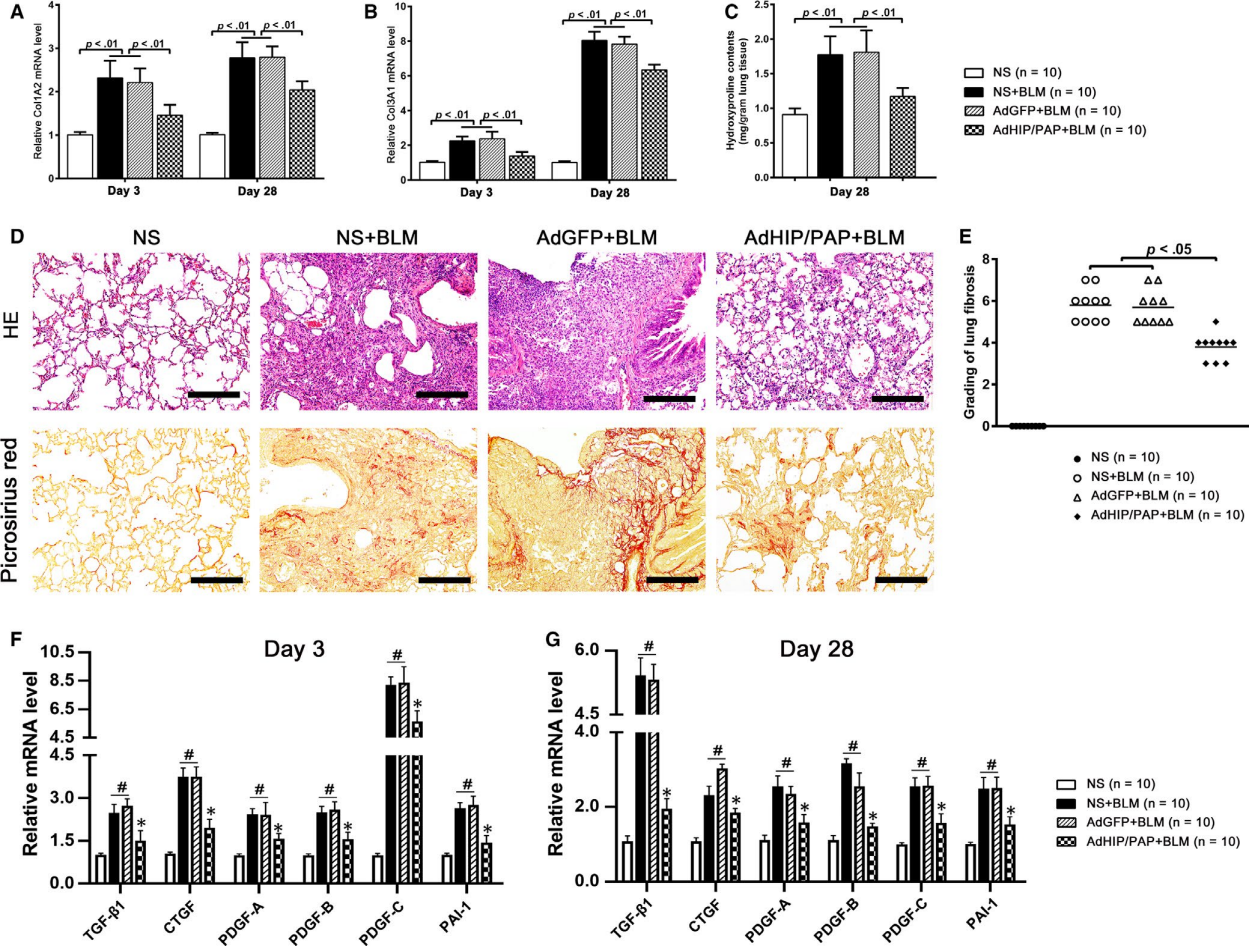
AdHIP/PAP alleviates BLM‐induced lung fibrosis in mice. The AdHIP/PAP‐mediated expression of HIP/PAP significantly attenuated BLM‐induced lung fibrogenesis in mice, as indicated by the decreased Col1A2 (A) and Col3A1 (B) mRNA expression, lower pulmonary hydroxyproline content (C), milder lung structure destruction, less picro‐sirius red‐positive collagen deposition (D) and, eventually, lower Ashcroft fibrosis score (E) compared with those of the NS + BLM and AdGFP + BLM groups. Moreover, overexpression of HIP/PAP lessened the BLM‐induced increases in pulmonary TGF‐β1; PDGF‐A, ‐B, and ‐C; CTGF; and PAI‐1 mRNA expression on both day 3 (F) and day 28 (G). ^#^ *P* < 0.05 vs the NS group, and ^*^ *P* < 0.05 vs the NS + BLM or AdGFP + BLM groups. Error bars indicate SD. Scale bars = 100 μm

Pulmonary fibrogenesis started as early as three days after BLM instillation, as indicated by the markedly increased pulmonary expression of Col1A2 and Col3A1 (Figure 4A,B). Twenty‐eight days after BLM treatment, the pulmonary hydroxyproline content was markedly increased (Figure 4C). H&E and picro‐sirius red staining followed by histological examination showed that normal lung architecture disappeared, lots of spindle‐shaped fibrotic cells clumped together, and bulky collagen fibers accumulated (Figure 4D), leading to a significantly increased fibrosis score (Figure 4E). Similar to the expression of Col1A2 and Col3A1, pulmonary TGF‐β1, platelet‐derive growth factor (PDGF)‐A, ‐B, ‐C, connective growth factor (CTGF) and plasminogen activator inhibitor (PAI)‐1 levels were markedly increased starting from day 3 (Figure 4F) following BLM treatment and were sustained until day 28 (Figure 4G). All the above fibrotic changes were significantly alleviated by AdHIP/PAP, while AdGFP showed no significant effect (Figure 4A‐G).

**FIGURE 5:**
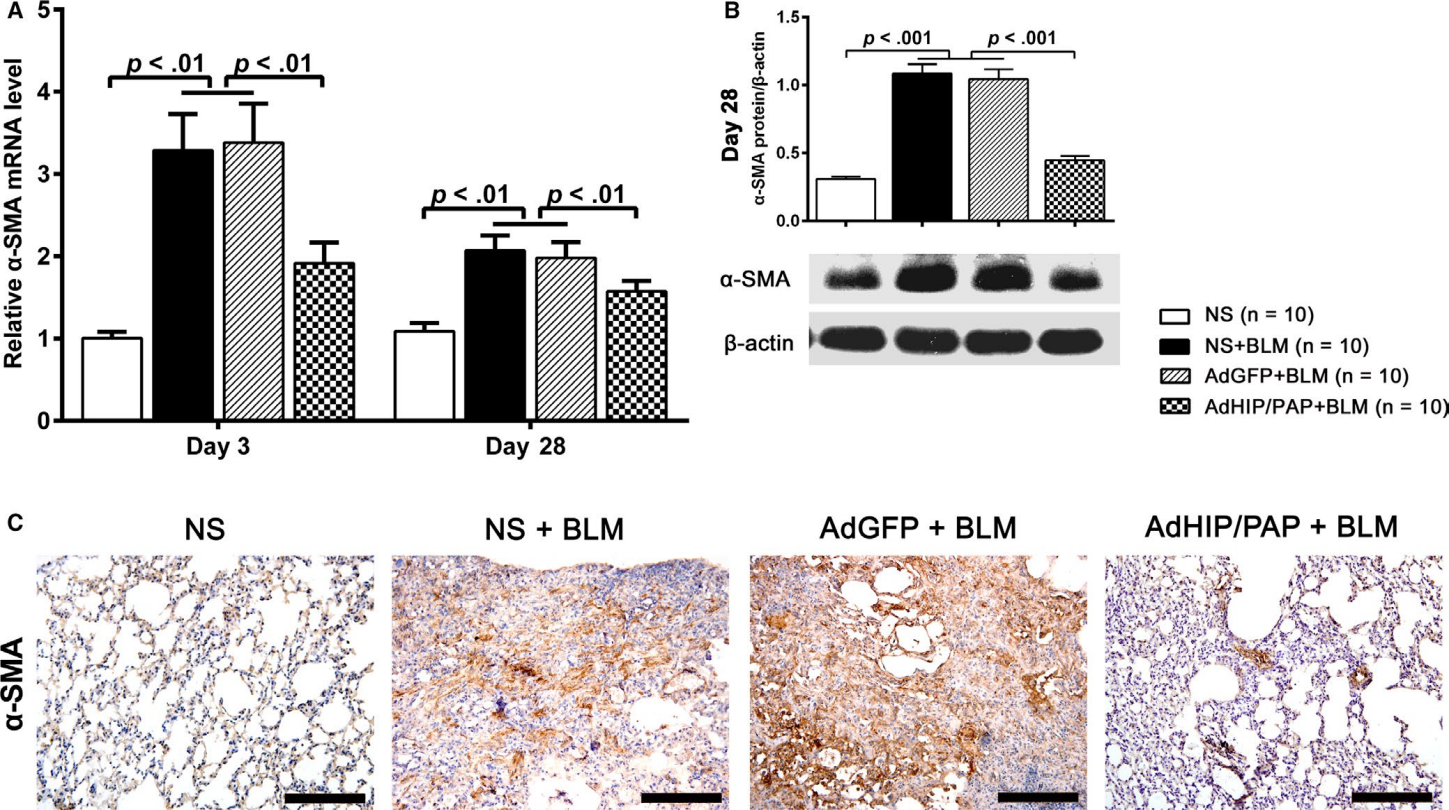
AdHIP/PAP inhibits BLM‐induced MFB activation in the mouse lung. AdHIP/PAP markedly alleviated the BLM‐induced excess expression of α‐SMA in the mouse lung tissue, as revealed by qRT‐PCR (A), western blotting (B) and immunohistochemistry (C), compared with those in the other two BLM groups (A‐C), indicating that HIP/PAP suppresses BLM‐induced MFB activation in mice. Error bars indicate SD. Scale bars = 100 μm

The expression level of α‐SMA, a marker for MFBs, in the AdHIP/PAP + BLM group was significantly lower than that in the other two BLM groups on both day 3 and day 28 (Figure 5A). The expression profile of α‐SMA on day 28 was also verified by western blotting (Figure 5B) and immunohistochemistry (Figure 5C).

### 3.5 HIP/PAP suppressed the proliferation and attenuated the TGF‐β1‐induced activation of HLF‐1 cells

**FIGURE 6:**
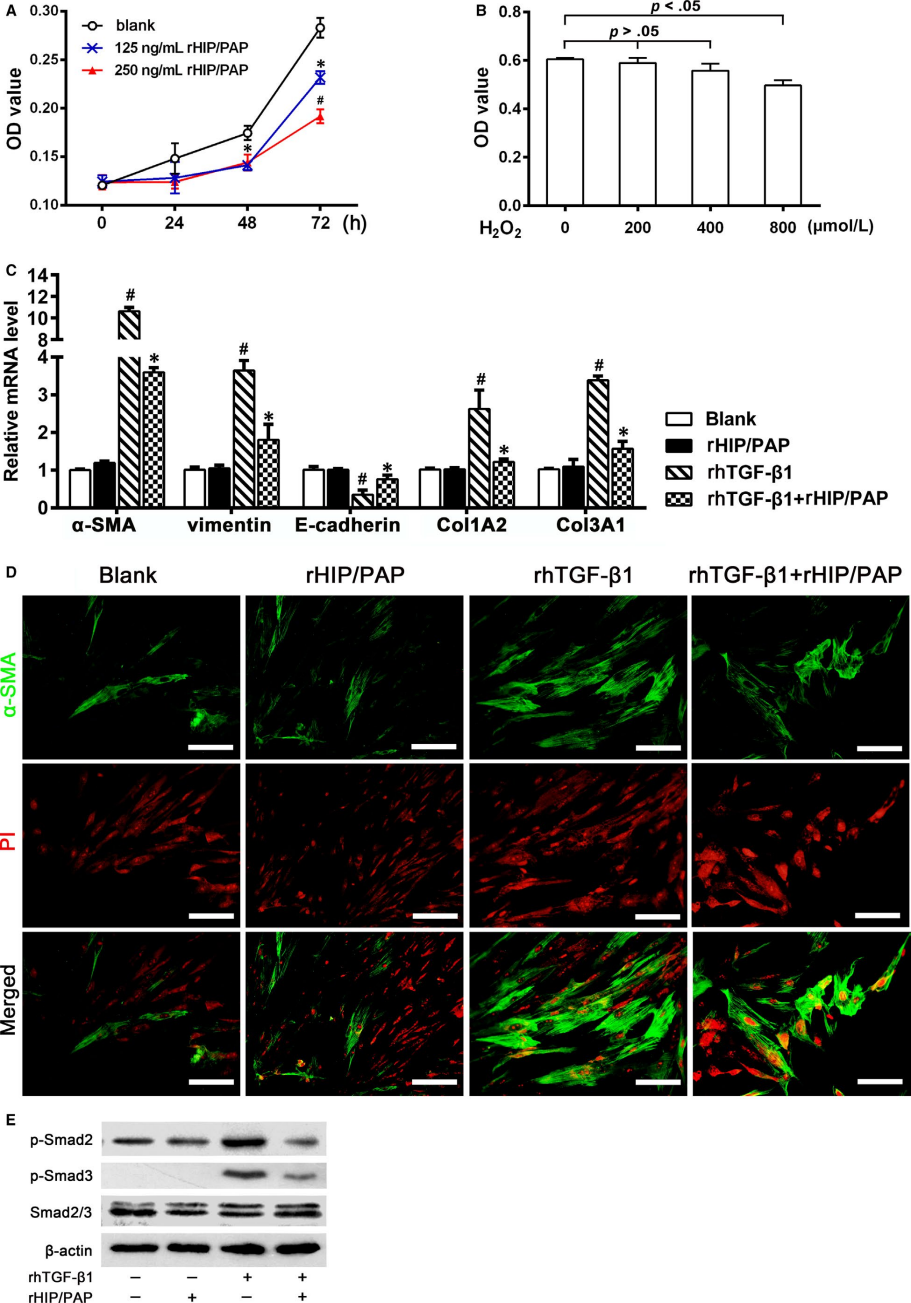
HIP/PAP suppresses proliferation and TGF‐β1‐induced activation in HLF‐1 cells. rHIP/PAP suppressed HLF‐1 cell growth in a concentration‐dependent manner. ^*^ *P* < 0.05 vs the blank control, ^#^ *P* < 0.05 vs 125 ng/mL rHIP/PAP (A). The cell viability assay showed that only when the concentration of H_2_O_2_ reached as high as 800 μM, could HLF‐1 viability be significantly affected, indicating that HLF‐1 cells are innately resistant to H_2_O_2_‐mediated oxidative injury (B). rHIP/PAP did not affect the basal expression of α‐SMA, vimentin, E‐cadherin, Col1A2, and Col3A1, but markedly attenuated the rhTGF‐β1‐induced upregulation of α‐SMA, vimentin, Col1A2, and Col3A1, as well as the downregulation of E‐cadherin in HLF‐1 cells. ^#^ *P* < 0.01, vs the blank or rHIP/PAP group, ^*^ *P* < 0.05 vs the rhTGF‐β1 group (C, D). Western blotting showed that rHIP/PAP abolished the rhTGF‐β1‐induced Smad2/3 phosphorylation in HLF‐1 cells, which further validated the antagonizing effect of rHIP/PAP on rhTGF‐β1 (E). Error bars indicate SD. Scale bars = 50 μm

The CCK‐8 assay showed that rHIP/PAP significantly inhibited the growth of HLF‐1 cells (Figure 6A). HLF‐1 cells presented distinct resistance to H_2_O_2_ injury. The viability of HLF‐1 cells was not significantly decreased until the H_2_O_2_ concentration was raised to 800 μmol/L (Figure 6B). qRT‐PCR revealed that rHIP/PAP did not significantly affect basal expression of α‐SMA, vimentin, and E‐cadherin, but markedly attenuated the rhTGF‐β1‐induced elevation of α‐SMA and vimentin and decreased expression of E‐cadherin in HLF‐1 cells (Figure 6C). The regulatory effect of rHIP/PAP on α‐SMA expression in HLF‐1 cells was further confirmed by immunofluorescence (Figure 6D). Similar results were also observed for Col1A2, Col3A1, CTGF, PDGF‐B, and TGF‐β1 expression in HLF‐1 cells (Figure 6C, Table 3). These results indicated that HIP/PAP exerted its functions, at least partly, by antagonizing TGF‐β1. Western blotting showed that rHIP/PAP remarkably mitigated rhTGF‐β1‐induced phosphorylation of Smad 2/3 but did not affect the basal levels of pSmad2/3 in HLF‐1 cells, confirming the antagonizing effect of HIP/PAP on TGF‐β1 (Figure 6E).

**TABLE 3:** HIP/PAP antagonizes the TGF‐β1‐induced expression of pro‐fibrotic factors in HLF‐1 and A549 cells and HPMVEC (relative mRNA levels)

	Blank	rHIP/PAP	rhTGF‐β1	rhTGF‐β1 + rHIP/PAP
HFL‐1				
TGF‐β1	1.01 ± 0.05	0.95 ± 0.08	3.01 ± 0.31^#^	1.83 ± 0.12*
CTGF	1.11 ± 0.10	1.09 ± 0.18	4.78 ± 0.13^#^	1.27 ± 0.08*
PDGF‐A	1.01 ± 0.03	1.01 ± 0.05	3.58 ± 0.53^#^	2.16 ± 0.29*
PDGF‐B	1.02 ± 0.03	0.97 ± 0.15	2.06 ± 0.20^#^	1.52 ± 0.14*
PDGF‐C	1.01 ± 0.05	0.93 ± 0.09	2.50 ± 0.08^#^	1.50 ± 0.14*
PAI‐1	1.01 ± 0.04	0.86 ± 0.15	7.31 ± 0.52^#^	3.72 ± 0.27*
A549				
TGF‐β1	1.00 ± 0.09	1.07 ± 0.10	2.37 ± 0.31^#^	1.56 ± 0.18*
CTGF	1.02 ± 0.03	1.11 ± 0.18	7.86 ± 0.18^#^	4.23 ± 0.25*
PDGF‐A	1.01 ± 0.06	0.96 ± 0.34	9.31 ± 1.17^#^	2.23 ± 0.29*
PDGF‐B	1.00 ± 0.04	1.16 ± 0.27	6.53 ± 0.78^#^	3.60 ± 0.51*
PDGF‐C	1.02 ± 0.06	1.11 ± 0.13	4.21 ± 0.22^#^	2.58 ± 0.16*
PAI‐1	1.02 ± 0.04	1.12 ± 0.16	11.05 ± 0.77^#^	6.69 ± 0.49*
HPMVEC				
TGF‐β1	1.02 ± 0.02	0.58 ± 0.12	0.62 ± 0.18	0.67 ± 0.15
CTGF	1.01 ± 0.04	1.13 ± 0.09	3.21 ± 0.31^#^	1.79 ± 0.19*
PDGF‐B	1.01 ± 0.02	0.97 ± 0.06	1.33 ± 0.08^#^	0.81 ± 0.12*
PDGF‐C	1.03 ± 0.07	0.98 ± 0.05	1.12 ± 0.08	1.09 ± 0.18
PAI‐1	1.04 ± 0.06	1.09 ± 0.12	3.42 ± 0.17^#^	3.02 ± 0.18

^#^ *P* < 0.01 vs the blank or rHIP/PAP groups, and

* *P* < 0.01 vs the rhTGF‐β1 group.

### 3.6 HIP/PAP promoted the proliferation of alveolar epithelial cells, protected them from H_2_O_2_‐induced apoptosis, and suppressed their TGF‐β1‐induced epithelial‐mesenchymal transition (EMT)

**FIGURE 7:**
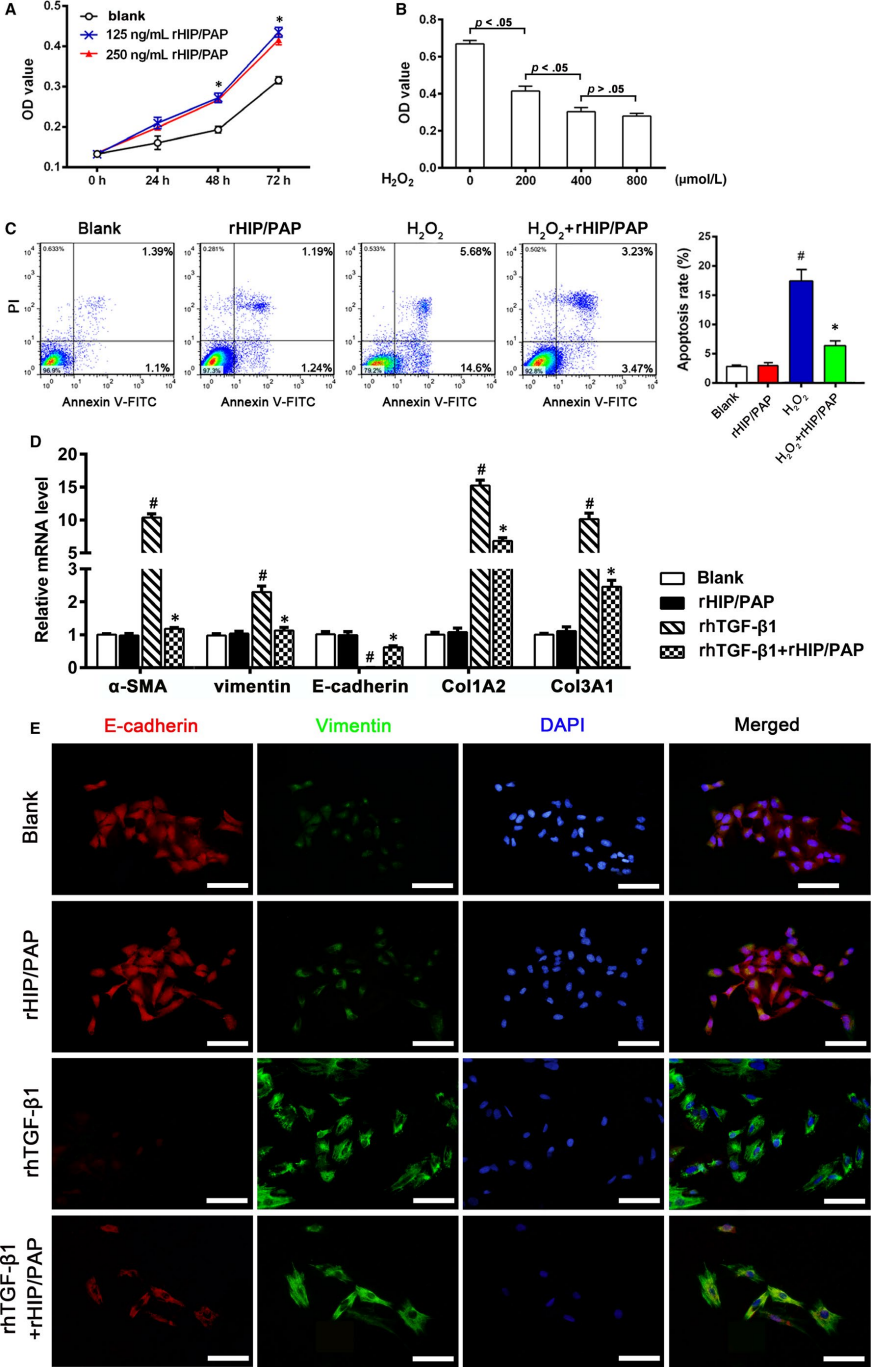
HIP/PAP promotes proliferation, alleviates H_2_O_2_‐induced apoptosis, and antagonizes TGF‐β1‐induced EMT in A549 cells. rHIP/PAP accelerated A549 cell growth. ^*^ *P* < 0.05 vs the blank control (A). H_2_O_2_ treatment suppressed A549 cell viability in a concentration‐dependent manner over the range of 0 ‐ 400 μmol/L (B). Flow cytometry analysis showed that rHIP/PAP (125 ng/mL) alleviated H_2_O_2_ (200 μmol/L)‐induced apoptosis in A549 cells. ^#^ *P* < 0.01, vs the blank or rHIP/PAP group, ^*^ *P* < 0.05 vs the H_2_O_2_ group (C). qRT‐PCR (D) and immunofluorescence (E) demonstrated that rHIP/PAP markedly abolished the rhTGF‐β1‐induced upregulation of α‐SMA, vimentin, Col1A2, and Col3A1 and the downregulation of E‐cadherin in A549 cells, while not affecting the basal expression of these molecules. ^#^ *P* < 0.01, vs the blank or rHIP/PAP group, ^*^ *P* < 0.05 vs the rhTGF‐β1 group. Error bars indicate SD. Scale bars = 50 μm

The effects of HIP/PAP on proliferation, H_2_O_2_‐induced apoptosis, and TGF‐β1‐induced EMT were tested in A549 and HPAEpiC. The CCK‐8 assay showed that rHIP/PAP significantly promoted the growth of A549 cells (Figure 7A). The H_2_O_2_ challenge markedly decreased the viability of A549 cells in a concentration‐dependent manner (Figure 7B), and rHIP/PAP markedly protected A549 cells from H_2_O_2_‐induced apoptosis (Figure 7C). qRT‐PCR showed that although rHIP/PAP did not affect the basal expression levels of E‐cadherin, vimentin, α‐SMA, Col1A2, and Col3A1, it markedly opposed the rhTGF‐β1‐induced upregulation of vimentin, α‐SMA, Col1A2, and Col3A1, as well as the rhTGF‐β1‐induced downregulation of E‐cadherin expression in A549 cells (Figure 7D). The opposing effect of rHIP/PAP on rhTGF‐β1‐induced EMT was further verified by immunofluorescence (Figure 7E). In addition, rHIP/PAP mitigated the rhTGF‐β1‐induced, but not the basal, expression of TGF‐β1, CTGF, PDGF‐B and ‐C, and PAI‐1 at the mRNA level (Table 3).

Moreover, rHIP/PAP exerted similar actions in HPAEpiC cells as in A549 cells, namely the acceleration of cell proliferation, the protection of cells from H_2_O_2_‐induced apoptosis, the inhibition of the rhTGF‐β1‐induced phenotype changes, and the inhibition of pro‐fibrotic cytokine expression (Figure S3).

### 3.7 HIP/PAP conferred accelerated proliferation, tolerance to H_2_O_2_‐induced apoptosis, and resistance to TGF‐β1‐induced endothelial‐mesenchymal transition (EndoMT) on human pulmonary microvascular endothelial cells

**FIGURE 8:**
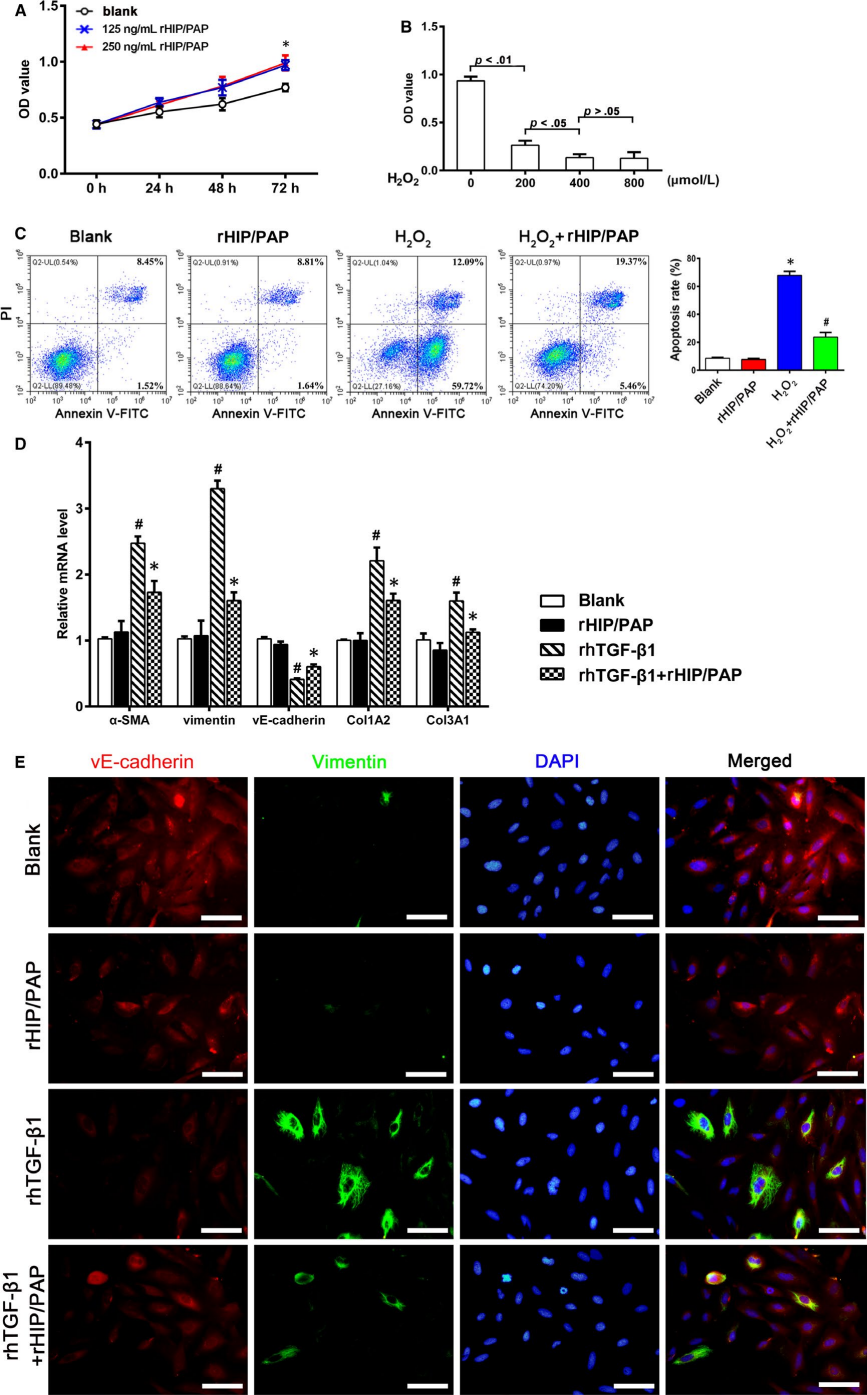
HIP/PAP accelerates proliferation, alleviates H_2_O_2_‐induced apoptosis, and inhibits TGF‐β1‐induced EndoMT in HPMVEC. rHIP/PAP at 125 and 250 ng/mL promoted HPMVEC growth to similar extents. ^*^ *P* < 0.05 vs the blank control (A). H_2_O_2_ treatment suppressed HPMVEC viability in a concentration‐dependent manner over the range of 0 ‐ 400 μM (B). Flow cytometry analysis revealed that rHIP/PAP (125 ng/mL) protects HPMVEC from H_2_O_2_‐induced (200 μM) apoptosis. ^*^ *P* < 0.01 vs the blank or rHIP/PAP group, ^#^ *P* < 0.05 vs the H_2_O_2_ group (C). rhTGF‐β1 (5 ng/mL) increased α‐SMA, vimentin, Col1A2, and Col3A1 expression and decreased vE‐cadherin expression in HPMVEC, while rHIP/PAP (125 ng/mL) markedly attenuated these alterations without affecting their basal expression (D, E). This suggests an antagonizing effect of HIP/PAP on the TGF‐β1‐induced EndoMT. ^#^ *P* < 0.01 vs the blank or rHIP/PAP group, ^*^ *P* < 0.05 vs the rhTGF‐β1 group. Error bars indicate SD. Scale bars = 50 μm

Pulmonary microvascular injury and EndoMT significantly contribute to PF. The CCK‐8 assay showed that rHIP/PAP accelerated HPMVEC growth (Figure 8A) and that HPMVEC were sensitive to H_2_O_2_‐induced oxidative injury (Figure 8B). Flow cytometry demonstrated that 200 nmol/L H_2_O_2_ induced significant apoptosis in HPMVEC, while 125 ng/mL rHIP/PAP significantly rescued HPMVEC from H_2_O_2_‐induced apoptosis (Figure 8C). Importantly, rHIP/PAP alleviated the rhTGF‐β1‐induced the upregulation of mesenchymal markers (vimentin and α‐SMA) and collagen synthesis and the downregulation of vE‐cadherin, a marker for microvascular endothelium, in HPMVEC (Figure 8D,E). In addition, rHIP/PAP abolished the TGF‐β1‐induced overexpression of CTGF and PDGF‐B in HPMVEC cells (Table 3). These results clearly indicated that HIP/PAP confers resistance to oxidative injury and TGF‐β1‐induced EndoMT in HPMVEC.

## 4 DISCUSSION

The present study demonstrated that HIP/PAP alleviated BLM‐induced lung injury, inflammation, and subsequent fibrosis in mice, suggesting a protective role of HIP/PAP in the pathogenesis of PF. Moreover, our data indicated that the protective effect of HIP/PAP may involve attenuating oxidative injury, promoting the regeneration of alveolar epithelial cells, and antagonizing the pro‐fibrotic actions of the TGF‐β1/Smad signaling pathway.

The lung is susceptible to high oxygen tension. Exogenous oxidants and pollutants can increase oxidant production in the lung. Under inflammation conditions, infiltrated and activated inflammatory cells, such as neutrophils, monocytes/macrophages, and eosinophils, can generate reactive oxygen species (ROS) via multiple enzymes/reaction pathways, including nicotinamide adenine dinucleotide phosphate oxidases, eosinophil peroxidase, and especially MPO.^25^, ^26^ MPO catalyzes the formation of potent cytotoxic oxidants, and the reaction of MPO with H_2_O_2_ can lead to the formation of peroxynitrite.^27^ Under normal physiological conditions, tissue has a complex and effective anti‐oxidant system to protect it from oxidative damage. Reduced glutathione (GSH, L‐γ‐glutamyl‐L‐cysteinyl‐glycine) is the most abundant non‐protein thiol in mammalian cells and acts as a reducing agent and a major antioxidant within cells by maintaining tight control of the redox status.^28^, ^29^ SOD decomposes superoxide radicals to H_2_O_2_. EC‐SOD, one kind of SOD isoform, is highly expressed in the lung and exerts its anti‐fibrotic effects, in part by preventing oxidative degradation of the extracellular matrix (ECM) and the release of ECM degradation products that can augment fibrosis. Overwhelming oxidative stress causes an oxidant‐antioxidant imbalance, results in widespread tissue destruction, and critically contributes to the development of PF.^25^, ^26^, ^27^, ^28^, ^29^, ^30^ Increased levels of BALF MPO^26^, ^30^ and oxidized protein^31^ were observed in idiopathic pulmonary fibrosis (IPF) patients, indicating that fibrotic lungs are under an increased oxidant stress. Moreover, BALF anti‐oxidants, such as SOD and glutathione S‐transferase P (GSTP), are downregulated in systemic sclerosis with PF compared with systemic sclerosis without PF.^31^ IPF patients exhibit glutathione deficiency in the epithelial lining fluid of the lower respiratory tract.^26^, ^30^ Increased oxidants were associated with epithelial injury in PF,^28^, ^29^ while reduced protein oxidation by ectopic glutaredoxin expression attenuated TGF‐β1 or BLM‐induced PF in mice.^32^ An interesting finding in our present study was that lung fibroblasts exhibited much stronger tolerance to H_2_O_2_‐induced apoptosis compared to alveolar epithelial cells, suggesting that lung fibroblasts might better survive oxidative stress and subsequently transform into extracellular matrix producing MFBs. In contrast, alveolar epithelial cells and pulmonary endothelial cells were much more susceptible to oxidative injury than pulmonary fibroblasts, implying that alveolar epithelial cells and pulmonary endothelial cells undergo more severe injury under the same level of oxidative stress. Alveolar epithelial injury leads to the impairment of air exchange function and, more importantly, the release of damage‐associated molecular patterns (DAMPs), such as HMGB1, ATP, mitochondrial DAMPs, and DNA, which can further initiate and sustain inflammation and fibrosis.^33^ Pulmonary microvascular injury and endothelial cell necrosis are present and proposed to be involved in IPF.^34^, ^35^

In the present study, we observed that HIP/PAP significantly attenuated BLM‐induced elevation of mouse pulmonary MPO activity and MDA content in vivo and protected alveolar epithelia and pulmonary microvascular endothelia from H_2_O_2_‐induced apoptosis in vitro, indicating an anti‐oxidative effect of HIP/PAP. Although the anti‐oxidative effect of HIP/PAP has been shown in previous studies,^17^, ^18^, ^20^, ^21^, ^22^ the underlying mechanism(s) has not been elucidated. A previous study showed that recombinant HIP/PAP is able to scavenge superoxide and hydroxyl free radicals in vitro;^22^ however, considering the amount, the extent to which this mechanism can contribute to the anti‐oxidative efficiency of HIP/PAP is questionable. Besides, Lieu et al^17^ and Moniaux et al^22^ have proposed that HIP/PAP might exhibit a superoxide dismutase‐like or glutathione reductase‐like activity, which could explain its protective effect against reactive oxygen species‐induced mitochondrial damages, subsequent cytochrome c release, and caspase‐3 activation induced by acetyl‐para‐aminophenol (APAP) overdose. However, our study did not show HIP/PAP (or Reg3B) to possess any SOD‐like activity. An interesting finding of our study was that HIP/PAP (and Reg3B) directly increased SOD expression in mouse lung tissue and cultured A549 and HLF‐1 cells, which might explain, to a large extent, how HIP/PAP (and Reg3B) exerted its anti‐oxidative effect on tissues and cells. Still, other possible mechanisms by which HIP/PAP exerted anti‐oxidative function cannot be excluded.

Although debates exist, inflammation is believed to contribute to the pathological progress of lung fibrosis, especially during the initial period.^36^ Infiltration of leukocytes into the lung leads to epithelial and endothelial cell dysfunction and tissue damage, which trigger fibrogenic processes, resulting in the deposition of matrix and lung structure remodeling.^36^ Moreover, infiltrated leukocytes and damaged tissue cells can release pro‐inflammatory cytokines, such as IL‐1β,^37^, ^38^ TNF‐α,^8^, ^9^, ^10^ IL‐6,^39^ IL‐17A,^40^ and HMGB1,^33^, ^41^ which also exert fibrogenic effects. Similarly, our present study demonstrated that HIP/PAP alleviated BLM‐induced lung inflammation as well as the production of pro‐fibrotic cytokines, suggesting that its anti‐inflammatory potency contributed to its anti‐fibrotic effect.

Besides protecting lungs from oxidative injury, our study demonstrated that HIP/PAP promotes the proliferation of alveolar epithelial cells and pulmonary microvascular endothelial cells. The mitogenic effect of HIP/PAP has been well identified in several tissues and cells.^17^, ^18^ The proliferation‐promoting effect of HIP/PAP might facilitate the repair of damaged alveoles and the pulmonary microvascular system to avoid aberrant repair leading to fibrosis. Contrary to its promoting effect on the proliferation of alveolar epithelial cells and pulmonary microvascular endothelial cells, HIP/PAP suppressed the growth of pulmonary fibroblasts, which should inhibit the accumulation of MFBs and further alleviate fibrogenesis.

In addition to the abovementioned findings, our in vivo and in vitro experiments demonstrated that HIP/PAP suppresses fibroblast activation, the EMT of alveolar epithelia, and the EndoMT of pulmonary endothelia by antagonizing TGF‐β1 signaling. MFBs are the major ECM‐producing cells in fibrotic lung tissue and are mainly derived from the activation/proliferation of resident lung fibroblasts, the EMT of alveolar and bronchiole epithelial cells, the EndoMT of microvascular endothelial cells, and the recruitment of circulating fibroblastic stem cells (fibrocytes).^35^, ^42^, ^43^, ^44^ TGF‐β1 has been verified as a pivotal pro‐fibrotic cytokine in that it can directly activate fibroblasts, induce EMT and EndoMT, promote ECM synthesis, inhibit ECM degradation and augment fibrosis by upregulating itself and other pro‐fibrotic factors, such as PDGF‐A and ‐B and CTGF.^45^ In the present study, we found that HIP/PAP attenuated BLM‐induced MFB formation and overexpression of TGF‐β1; CTGF; PDGF‐A, ‐B, and ‐C; and PAI‐1 in mouse lungs. rHIP/PAP suppressed the TGF‐β1‐induced activation of lung fibroblasts, EMT of alveolar epithelial cells, and EndoMT of pulmonary microvascular endothelial cells, as well as reduced the TGF‐β1‐induced upregulation of TGF‐β1; CTGF; PDGF‐A, ‐B, and ‐C; and PAI‐1 in these cells. These results demonstrated that abolishing the pro‐fibrotic effects of TGF‐β1 is one of the critical mechanisms through which HIP/PAP alleviated BLM‐induced PF. Moreover, the TGF‐β1‐antagonizing effect of HIP/PAP was also verified in our recent study on CCl_4_‐induced mouse liver fibrosis.^46^

In summary, we found here, that the increased pulmonary production of HIP/PAP presents an adaptive/compensatory reaction for lung fibrosis. Pulmonary overexpression of HIP/PAP mediated by recombinant adenovirus efficiently ameliorated BLM‐induced murine lung injury and fibrosis via multiple activities, suggesting a protective role of HIP/PAP in PF.

## CONFLICT OF INTEREST

The authors confirm that they have no conflicts of interest.

## AUTHORS' CONTRIBUTIONS

HZ and ZX conceived and designed the research; ZX, TH, LQ and WY carried out experiments; LY prepared the adenoviruses; HY, ZX and LH analysed the data; ZX and LQ drafted the manuscript; HZ, HY and HL revised the manuscript; all authors approved the final manuscript.

## Supporting information

Supplementary Material

## Data Availability

The data that supports the findings of this study are available in the supplementary material of this article.
