# Supplementary material for: HIP/PAP protects against bleomycin‐induced lung injury and inflammation and subsequent fibrosis in mice
Source: J Cell Mol Med. 2020 Apr 30;24(12):6804–21. doi: 10.1111/jcmm.15334 (PMC7299702; doi:10.1111/jcmm.15334)
Supplement: Supplementary file 1 — Supplementary Material [file JCMM-24-6804-s001.pdf]

Supplementary Table S1. Clinical data and pathological features of the human lung tissue specimens.

Pathological diagnosis	n
Normal controls (6 males, 4 females, aged 52.20 ± 13.43 yrs)*	
Normal lung tissue	2
Cancer-adjacent normal lung tissue	8
Fibrotic lung tissues (7 males, 3 females, aged 58.20 ± 12.47 yrs)*	
Cancer-adjacent pulmonary interstitial fibrosis	4
Cancer-adjacent chronic interstitial pneumonia with interstitial fibrosis	2
Chronic bronchitis with pulmonary interstitial fibrosis	2
Cancer-adjacent pulmonary collapse with interstitial fibrosis	1
Cancer-adjacent chronic interstitial pneumonia with interstitial fibrosis	1

*There were no significant differences in gender and age between the two groups ($P > 0.05$, Chi-square test).

Supplementary Table S2. Primers used in qRT-PCR

Gene		Primers
**Mouse β-actin**	**F**	5’-CATCCGTAAAGACCTCTATGCCAAC -3’
	**R**	5’-ATGGAGCCACCGATCCACA-3’
**Mouse Reg3B**	**F**	5’- GAACCCAATGGAGGTGGATG -3’
	**R**	5’- ATTTGCAGACATAGGGCAAC -3’
**Mouse CD45**	**F**	5’- TCAATCTCTTGGAAAGTGCAG-3’
	**R**	5’- TTCTTGACTTGTCCATTCTGG-3’
**Mouse Cu-Zn SOD**	**F**	5’- TGTGTCCATTGAAGATCGTG -3’
	**R**	5’- AATCACTCCACAGGCCAA-3’
**Mouse Mn-SOD**	**F**	5’- TCAATAAGGAGCAAGGTCGC-3’
	**R**	5’- TCTCCCAGTTGATTACATTCC-3’
**Mouse EC-SOD**	**F**	5’- TGGCCTGAACTTCACCAGAG-3’
	**R**	5’- GTCTGCTAGGTCGAAGCTGG-3’
**Mouse Col1A2**	**F**	5’-AGAGACTATCAATGGTGGCAGC-3’
	**R**	5’-TCTCCTCATCCAGGTACGCA-3’
**Mouse Col3A1**	**F**	5’- CACAGTTCTAGAGGATGGCTG -3’
	**R**	5’- CACACCAAATTCTTGATCAGGAC -3’
**Mouse TGF-β1**	**F**	5’- CATGCCAACTTCTGTCTGGGA -3’
	**R**	5’- CTTGCGACCCACGTAGTAGAC -3’
**Mouse CTGF**	**F**	5’-ACCCGAGTTACCAATGACAATACC-3’
	**R**	5’-CCGCAGAACTTAGCCCTGTATG-3’
**Mouse PDGF-A**	**F**	5’- AACACCAGCAGCGTCAAGTG -3’
	**R**	5’- TTCAGGTTGGAGGTCGCAC -3’
**Mouse PDGF-B**	**F**	5’- CTCTCGGAACCTCATCGATC -3’
	**R**	5’- GGCTTCTTTCGCACAATCTC -3’
**Mouse PDGF-C**	**F**	5’- TGGACTTGGACAGCCTCTAC -3’
	**R**	5’- TGTTCCAGAGCCACATCAG -3’
**Mouse PAI-1**	**F**	5’- AGGATCGAGGTAAACGAGAG -3’
	**R**	5’- GATCGGTCTATAACCATCTC -3’
**Mouse α-SMA**	**F**	5’-GCCAGAACTTCCCAACCAT-3’
	**R**	5’-TCAGAGCCCAGAATTTTCTCC-3’
**Human β-actin**	**F**	5’-CCACACCCGCCACCAGTTCG-3’
	**R**	5’-TACAGCCCGGGGAGCATCGT-3’
**Human α-SMA**	**F**	5’- CTGGCATTGCCGACCGAATG -3’

	**R**	5'- GATCCACATCTGCTGGAAGG -3'
**Human E-cadherin**	**F**	5'- GACTCGTAACGACGTTGCAC -3'
	**R**	5'- AGACTAGCAGCTTCGGAACC -3'
**Human vE-cadherin**	**F**	5'- GATGGAGACAGCAGGAGACT-3'
	**R**	5'- TGGATTGGAGCAGAACTCTG-3'
**Human vimentin**	**F**	5'- GATTCACTCCCTCTGGTTGA -3'
	**R**	5'- GCTGCACTGAGTGTGTGCAA -3'
**Human Col1A2**	**F**	5'-GAGGGCAACAGCAGGTTCACTTA-3'
	**R**	5'-TCAGCACCACCGATGTCCA-3'
**Human Col3A1**	**F**	5'- CACCTACACAGTTCTGGAGG -3'
	**R**	5'- CCACCAATGTCATAGGGTGC -3'
**Human TGF-β1**	**F**	5'-TCCTGGCGATACCTCAGCAA-3'
	**R**	5'-GCTAAGGCGAAAGCCCTCAA-3'
**Human CTGF**	**F**	5'-CTTGCGAAGCTGACCTGGAA-3'
	**R**	5'-AGCTCAAACTTGATAGGCTTGGAGA-3'
**Human PDGF-A**	**F**	5'-TGTGGCCAGCACACCAAGT-3'
	**R**	5'-AGGTCACCATCTACAGCCACCTC-3'
**Human PDGF-B**	**F**	5'- TGTGGCCAGCACACCAAGT -3'
	**R**	5'- AGGTCACCATCTACAGCCACCTC -3'
**Human PDGF-C**	**F**	5'- CCAGAAGATGACATATGCAAG -3'
	**R**	5'- TAAGTCCAACTGCCATCTCTC -3'
**Human PAI-1**	**F**	5'- ACCCTCAGCATGTTCATTGC -3'
	**R**	5'- TTCCTGAGGTCGACTTCAGT -3'

Supplementary Figure S1

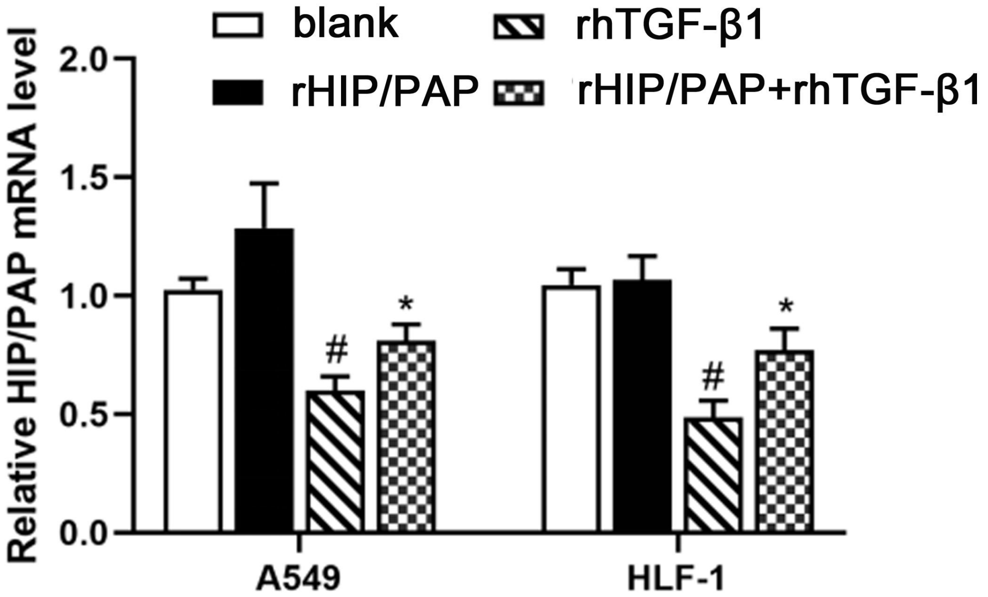

Supplementary Figure S1. The effect of TGF-β1 on HIP/PAP expression in A549 and HLF-1 cells. rhTGF-β1 (5 ng/mL) significantly inhibited HIP/PAP expression in both A549 and HLF-1 cells, while rHIP/PAP (125 ng/mL) markedly mitigated this effect. # $P < 0.01$ vs the blank or rHIP/PAP group * $P < 0.05$ vs the rhTGF-β1 group.

Supplementary Figure S2.

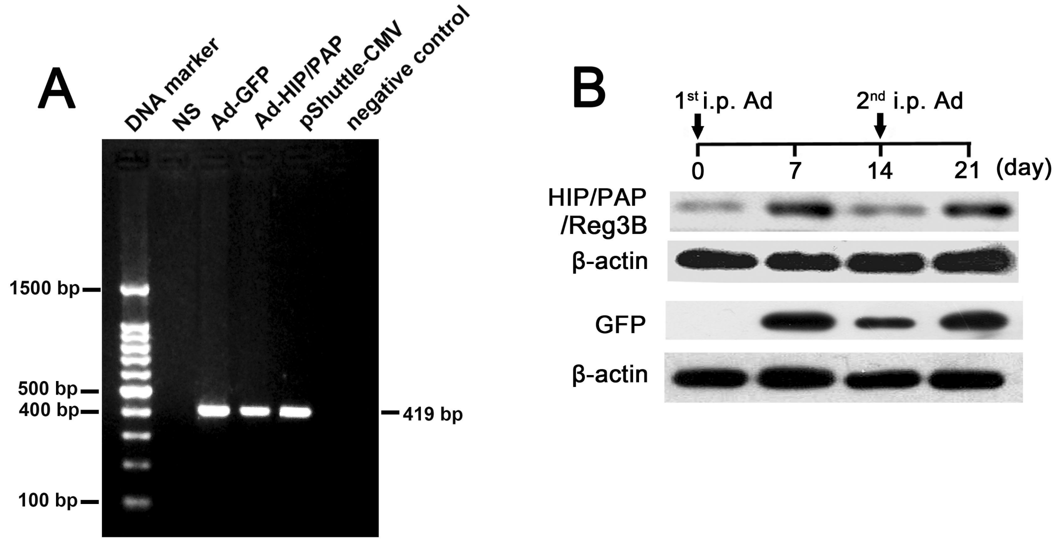

Supplementary Figure S2. Repeated intraperitoneal injection of recombinant adenovirus efficiently mediates foreign gene expression in mouse lung tissue. Mice were intraperitoneally administered twice, fourteen days apart, adenoviruses carrying HIP/PAP or GFP. The CMV-IE sequence was detected by PCR in mouse lung tissues seven days after intraperitoneal adenovirus administration, verifying the efficient transduction of the adenoviruses (A). Western blotting showed that the expression levels of HIP/PAP (Reg3B) and GFP following the second injection of the adenoviruses were comparable to those after the first injection of adenoviruses in mouse lung tissues (B).

Supplementary Figure S3

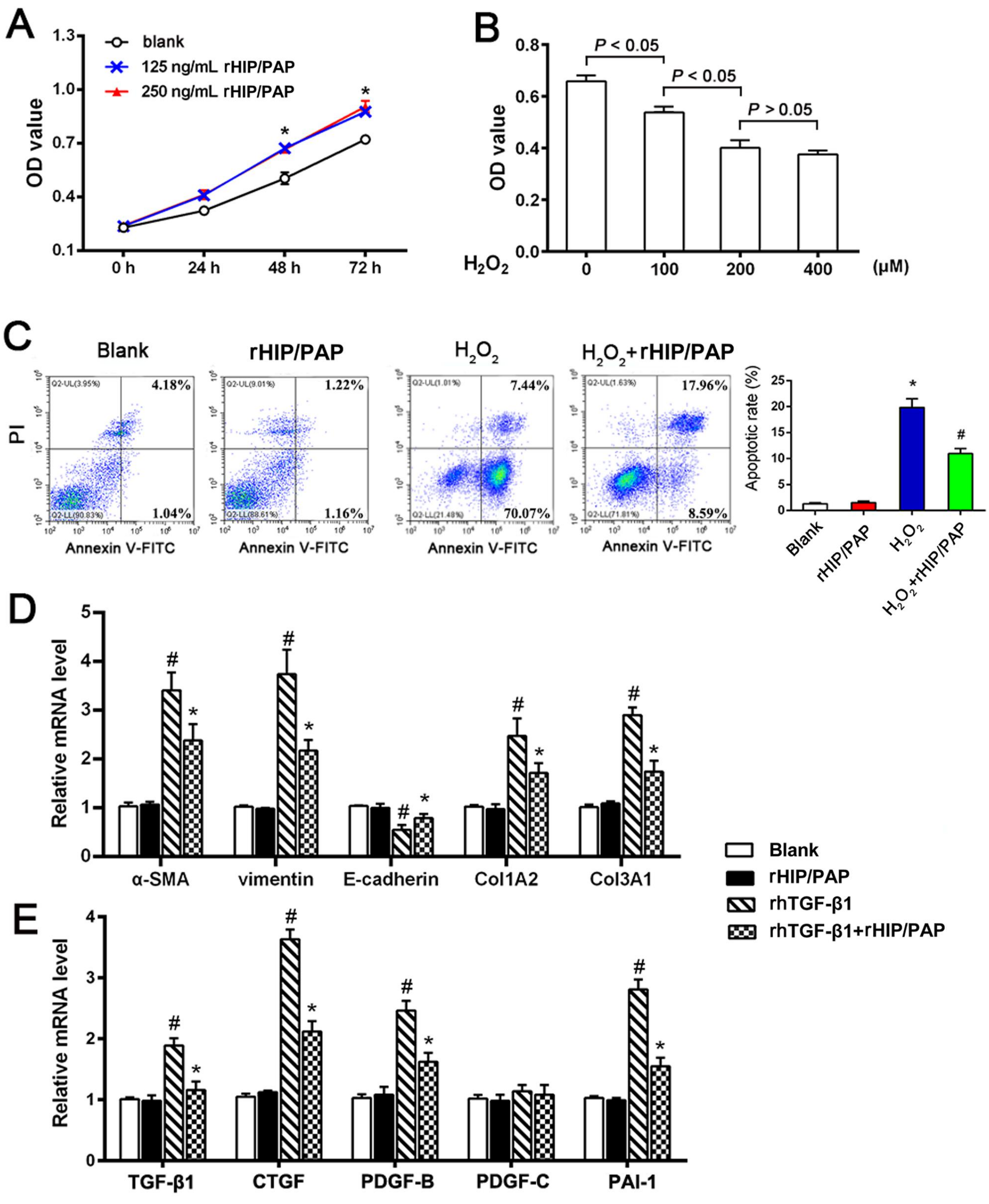

Supplementary Figure S3. HIP/PAP promotes proliferation, alleviates H_2O_2-induced apoptosis, and antagonizes TGF-β1-induced EMT in HPAEpiC cells. rHIP/PAP at 125 and 250 ng/mL accelerated the growth of HPAEpiC to similar extents. *$P < 0.05$ vs the blank control (A). H_2O_2 suppressed HPAEpiC viability in a concentration-dependent manner over the range of 0 —200 μM. * $P < 0.05$ vs the blank group (B). Flow cytometry analysis revealed that H_2O_2 at 200 μM induced apoptosis in HPAEpiC cells, which was significantly inhibited by rHIP/PAP (125 ng/mL). # $P < 0.05$ vs the H_2O_2 group, * $P < 0.01$ vs the blank or rHIP/PAP group (C). rHIP/PAP

(125 ng/mL) markedly abolished the rhTGF-β1-induced (5 ng/mL) upregulation of α-SMA, vimentin, Col1A2, Col3A1, TGF-β1, CTGF, PDGF-B, PAI-1 and the downregulation of E-cadherin in HPAEpiC at the mRNA level, indicating an inhibitory effect of rHIP/PAP on rhTGF-β1-induced EMT. * $P < 0.05$ vs the rhTGF-β1 group, # $P < 0.01$ vs the blank or rHIP/PAP group (D, E).
